# Editorial: Exercise for age-related musculoskeletal disorders

**DOI:** 10.3389/fpubh.2023.1337093

**Published:** 2023-12-27

**Authors:** Xi Chen, Kai Chen, Jun Zou

**Affiliations:** ^1^School of Sports Science, Wenzhou Medical University, Wenzhou, China; ^2^School of Molecular Sciences, University of Western Australia, Perth, WA, Australia; ^3^School of Kinesiology, Shanghai University of Sport, Shanghai, China

**Keywords:** exercise, musculoskeletal disorders, aging, rehabilitation, effect

With the aging of the global population, the prevalence of age-related musculoskeletal disorders has rapidly escalated, becoming a significant public health priority (1). Age-related musculoskeletal disorders encompass a diverse spectrum of pathological conditions that affect muscles, bones, cartilage, and various other bodily tissues, thereby resulting in substantial physical and functional limitations among affected individuals. These disorders include, but are not confined to, conditions such as sarcopenia, osteoporosis, osteoarthritis, intervertebral disc degeneration, rheumatoid arthritis, and ankylosing spondylitis (1, 2). Exercise has emerged as a pivotal and widely employed strategy for the prevention and treatment of musculoskeletal disorders. In response to health concerns, exercise or rehabilitation exercise regimens are meticulously tailored to enhance not only cardiorespiratory fitness but also flexibility, balance, strength, and power (3). Some key interventional studies have shown that exercise or physical activity improves muscle and skeletal disorders in aging people, especially sarcopenia and osteoporosis (4). In addition, research findings also demonstrated some of the molecular mechanisms that exercise or physical activity benefit skeletal and muscle health (5). However, more investigations are needed to further explore the effects of exercise on the prevention or rehabilitation of age-related muscle and skeletal disorders.

While extant research has elucidated the molecular mechanisms underlying the salutary impact of exercise and physical activity on skeletal and muscle health, further investigations are still necessary. Therefore, the Research Topic “*Exercise for age-related musculoskeletal disorders*” was launched to collect more articles on exercise and age-related musculoskeletal disorders, aiming to better understand the potential treatment of musculoskeletal disorders in the aging population. In this Research Topic, we divided the published articles into three key themes to provide a comprehensive overview of the role of exercise in age-related musculoskeletal challenges.

## Theme one: alleviating pain in aging

The first theme explored the efficacy of exercise in addressing attenuating pain associated with age-related musculoskeletal disorders. Osteoarthritis is one of the main musculoskeletal disorders causing knee pains in aging people. Zhang et al. conducted a meta-analysis and found that traditional Chinese exercises including Taijiquan, Baduanjin, Yijinjing, and Wuqinxi alleviated knee pain, stiffness, and improved the physical function in knee osteoarthritis patients. Among these exercises, Taijiquan exhibited more benefits for knee pain and dysfunction (Zhang et al.). Another network meta-analysis explored exercise interventions for chronic low back pain, and it revealed that exercise interventions improved pain in low back pain patients, and Taijiquan was even better than conventional rehabilitation (Li et al.). These findings suggest that exercise serves as a promising intervention for alleviating pain in the aging population.

## Theme two: diverse approaches to exercise for older adults

The second theme illuminated a diverse array of exercise modalities specifically tailored to older adults. A bibliometric analysis conducted by Mi et al. revealed the research trends in resistance training for aging individuals. The bursts of co-occurrence keyword map highlight the key terms such as “insulin-like growth factor expression,” “blood pressure,” and “health-related quality” are associated with resistance training in aging individuals, which provides valuable insights into research hotspots exercise for improving chronic diseases such as diabetes and hypertension in older adults. Additionally, physical function also is the research frontier in the field of resistance training. Zhou et al. provided us better understanding of pelvic floor muscle exercise may benefit to improving urinary continence following surgery, especially 3 months after radical prostatectomy. Furthermore, Alizadeh et al. contributed to the theme by sharing teleexercise training for geriatric patients with failed back surgery syndrome, providing the potential utility of telehealth platforms for further health care in aging.

## Theme three: mechanisms unveiled

This theme mainly unveiled the mechanisms of exercise to combat aging-related musculoskeletal disorders. Mitochondrial dysfunction is accepted as a key role in the pathogenesis of conditions, such as sarcopenia, in the aging population. A comprehensive article revealed the intricate relationship between mitochondrial dysfunction and sarcopenia, a chronic degenerative disease that affects older adults. It outlines how exercise can effectively address mitochondrial dysfunction, promoting mitochondrial health and reducing the impact of aging on the musculoskeletal system (Zhu et al.). Older adults are at higher risk of falls, Liu Z. et al. investigated the interplay between lower extremity muscle strength, proprioception, and tactile sensation in maintaining postural stability in aging, and found that sensory deficits are a significant risk factor for falls among older adults. Moreover, another article showed that a Chinese traditional exercise—Yi Jing Bang (YJB) exercise has more upward rotation and a similar or less anterior tilt than the mean resting scapular angle, which provides insights into the potential of YJB exercises in shoulder rehabilitation (Liu J. et al.).

## Conclusion

In conclusion, the compilation of eight articles significantly enhanced our comprehension of exercise interventions for age-related musculoskeletal disorders, promising improved quality of life for older adults. However, we must acknowledge that further research and exploration are required to validate and refine exercise intervention as a powerful tool for addressing age-related musculoskeletal disorders in the aging population.

## Author contributions

XC: Writing—original draft, Writing—review & editing. KC: Writing—review & editing. JZ: Writing—review & editing.
